# Automating dChip: toward reproducible sharing of microarray data analysis

**DOI:** 10.1186/1471-2105-9-231

**Published:** 2008-05-08

**Authors:** Cheng Li

**Affiliations:** 1 Department of Biostatistics and Computational Biology, Dana-Farber Cancer Institute. Department of Biostatistics, Harvard School of Public Health. 44 Binney St. Boston, MA 02115, USA

## Abstract

**Background:**

During the past decade, many software packages have been developed for analysis and visualization of various types of microarrays. We have developed and maintained the widely used dChip as a microarray analysis software package accessible to both biologist and data analysts. However, challenges arise when dChip users want to analyze large number of arrays automatically and share data analysis procedures and parameters. Improvement is also needed when the dChip user support team tries to identify the causes of reported analysis errors or bugs from users.

**Results:**

We report here implementation and application of the dChip automation module. Through this module, dChip automation files can be created to include menu steps, parameters, and data viewpoints to run automatically. A data-packaging function allows convenient transfer from one user to another of the dChip software, microarray data, and analysis procedures, so that the second user can reproduce the entire analysis session of the first user. An analysis report file can also be generated during an automated run, including analysis logs, user comments, and viewpoint screenshots.

**Conclusion:**

The dChip automation module is a step toward reproducible research, and it can prompt a more convenient and reproducible mechanism for sharing microarray software, data, and analysis procedures and results. Automation data packages can also be used as publication supplements. Similar automation mechanisms could be valuable to the research community if implemented in other genomics and bioinformatics software packages.

## Background

During the past decade, many microarray software packages have been developed for data analysis and visualization of gene expression, comparative hybridization, and tiling microarrays. Among these packages are Cluster/TreeView, GenePattern, GenMAPP, BRB ArrayTools, GSEA, SAM, TM4, GeneSpring and dChip. In addition, Bioconductor [1] builds on the R environment to provide programming capabilities and libraries for analyzing microarray and genomics data. These software packages have greatly contributed to translating large, raw datasets into testable biological hypotheses. In particular, we have developed and maintained dChip as a microarray software package accessible to both biologists and data analysts through a friendly user interface [2,3]. dChip has been widely used for expression and SNP (single nucleotide polymorphism) microarray data analysis, due to its many data-processing functions and interactive exploration views for probe-level, clustering, and chromosome-level data.

From our experiences with dChip software development, user support, and data analysis, we noticed several related issues. First, users or microarray core consultants often share their dChip analysis procedures with colleagues. However, the second user must manually follow the exact menu steps and analysis parameters to achieve the same results or see the same data view as the first user. This process is time-consuming and error-prone. Second, when dChip analysis errors or potential bugs occur, the information provided by users is often incomplete and out of context. To provide solutions or debug code, we require more information, which often is obtained through iterations of messages before we are able to recreate the error scenario using our own data. Third, routine dChip operations on new datasets could better be automated with minimal user intervention.

We report here the implementation a dChip automation module to meet these challenges. Using this module, dChip automation files can be interactively created when a user performs data analyses. These files contain menu steps with their parameters stored in a set of parameter files, as well as viewpoints that record particular positions and image sizes in clustering or chromosome views. A data-packaging function can let one user conveniently send microarray data, automation parameter files, and the dChip executable file to a recipient. On the recipient's computer, dChip can automatically follow the exact menu steps and parameters to recreate the entire analysis session, such as generating an analysis output or going to particular chromosome positions. An analysis report file can also be generated during an automated run, including an analysis log, user comments, and viewpoint screenshots.

There are other automation approaches in software packages or web services for genomics analysis. Taverna is a software tool for designing and executing workflows [4], and it allows user to integrate various tools or web services such as NCBI or Bioconductor. Taverna conveniently organizes and visualizes analysis steps in a flow chart, and it also provides a repository of workflows that can be shared among users. Using the R statistical environment, Gentleman and colleagues have proposed reproducible microarray data analysis via a compendium consisting of codes, data, and manuscript texts [5]. In addition, microarray and proteomics data standards such as MIAME, MAGE and MIAPE have greatly facilitated sharing of datasets among researchers using different software packages. Comparing to these tools, the dChip automation module has a minimal learning curve (the existing dChip users can easily automate and share their analysis), does not require user programming, and emphasizes the reproducible sharing of analysis data and procedures among dChip users. Through the dChip automation module, we also intend to illustrate simple implementation principles that can be adopted by other genomics software packages, which when automated could be utilized in a workflow environment such as Taverna.

## Implementation and examples

### Creating automation steps or viewpoints

**Figure 1:**
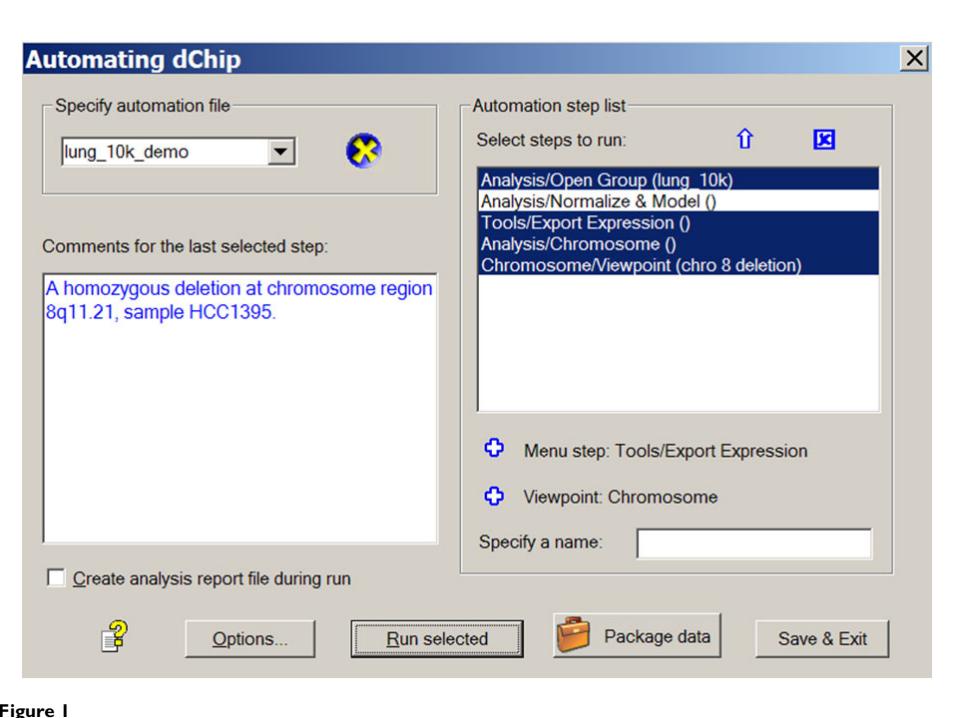
**The dChip automation dialog to specify automation file name, organize menu steps and viewpoints, and edit comments.** The following menu steps and viewpoints are included in the automation file "lung_10k_demo": "Analysis/Open group" menu to open a set of array image files and read in a sample information file, "Analysis/Normalize & Model" menu to normalize data and compute SNP signal values, "Tools/Export expression" menu to export signal values into a data file, "Analysis/Chromosome" menu to view copy number and loss of heterozygosity data along chromosome, and a chromosome viewpoint to display an interesting chromosome region. Comments associated with an automation step can be edited in the box on the left. "Analysis/Normalize & Model" is not selected in the dialog since it only needs to be run once to save results into dChip internal data files, which are used in subsequent or shared dChip sessions.

An "Automation" dialog was implemented to let a user create automation steps (Figure 1). After a menu step is manually executed, it is available in this dialog and can be added to the "Automation step list." Most menu items in dChip to import or export data or perform analysis functions have been included in this automation scheme. In addition, a user can interactively zoom to a gene cluster or a chromosome region and record the corresponding viewpoint through step-parameter files. Names and comments can be associated with each menu step or viewpoint to note particular findings or analysis settings. The menu steps selected are checked for internal coherence before saving to an automation file, ensuring a menu step such as "Analysis/Chromosome" is executed after "Analysis/Open group." Automation files are saved in the XML format for compatibility between different file versions and for human readability.

### Storing step settings

We implement the automation function based on dChip's existing setting files (with *.ini extension). A setting file stores the analysis parameters and settings of a dChip session when analyzing a group of microarray images or an external data file. The parameters include the data directory name, normalization and signal summarization parameters, and clustering and chromosome view settings. When a user starts dChip to resume a previous analysis, the session name used (the same as the setting file name) can recall the stored parameters so the same or a modified analysis can be performed. Because a setting file stores only the most recent parameters used before exiting dChip and parameters can change during an analysis session, we provide the option to save a named setting file for each menu step or viewpoint as they are added, so that we can recreate the entire analysis session. These setting files capture the current status of many dChip parameters, such as the file name of a gene list used for clustering, image size for a data point, and the current gene or SNP to be centered in a chromosome view. The setting files are saved in the current working directory.

### Running automated steps

**Figure 2:**
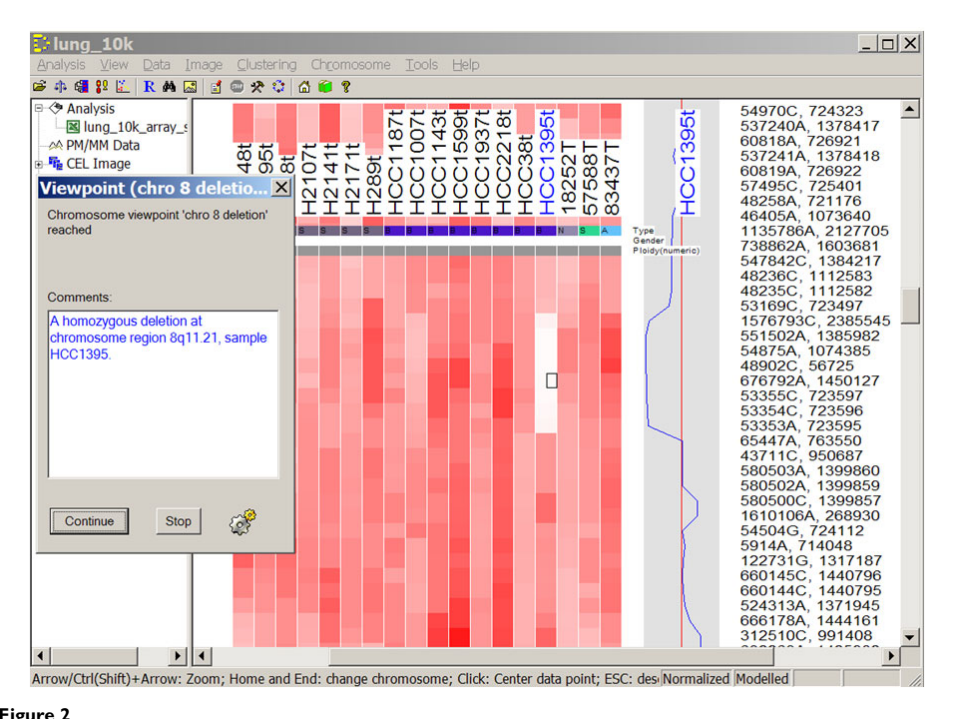
**A chromosome viewpoint is reached during an automated run.** A dialog displays the user comments for this viewpoint.

**Figure 3:**
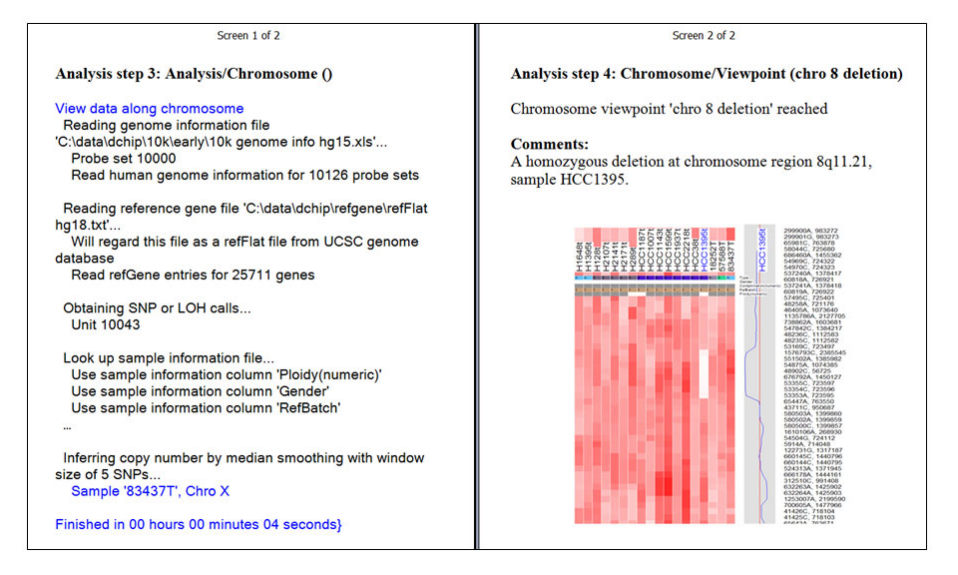
Part of the analysis report file generated after an automated run, viewed in the Microsoft Word "Reading" layout.

The specified menu steps or viewpoints can be selected to run sequentially and automatically. We use one dataset to illustrate an automated run. The automation data package can be downloaded from the project homepage (see below). It includes a set of 10 K SNP arrays used to study chromosome alterations in lung cancer samples [6]. At the automation dialog (Figure 1), the "Run selected" button can be clicked to run the selected steps sequentially. The stored parameters in the setting files will be loaded before running each step, in lieu of the traditional dChip dialogs asking for users to specify parameter values. Alternatively, if an automation file has been saved and specified, on a new dChip start, dChip will automatically load the automation file and run the steps. If a viewpoint is reached or a menu step has associated comments, the automated run will be paused while dChip shows the current data view and a message box displays comments (Figure 2). The user can choose to continue the automated run or to stop and explore the current data view interactively and then use the "Tools/Automate" dialog to resume the run. These menu steps enable another user to recreate the entire analysis from original array image files. An analysis report file in Microsoft Word format can also be automatically saved to include an analysis log, user comments, and clustering or chromosome images of viewpoints (Figure 3). This file contains an overview of the results from the automated steps and can be used as a preliminary analysis report.

### Packaging data and sharing analysis

The above procedures are very useful for a single user to automate frequently executed menu steps or recreating interesting data viewpoints. However, a much more beneficial application is to share these automate steps or viewpoints with local or remote colleagues. Traditional sharing of results and procedures with colleagues involves transferring data tables and figures as individual files or including them in a report file, along with descriptions explaining the steps used and specified parameters. Sometimes, images need to be prepared in various sizes to have both a global view as well as focused views of specific image regions. In the new dChip automation module, we have implemented a "Package data" function. This function copies all of the needed data, the automation and setting files used, and the dChip software (a 2-Mb executable file) into a new directory. This new data directory can then be transferred to another colleague's computer. When running the dChip executable file, the exact analysis steps, viewpoints, and comments are reproduced. The user can pause at any step to interactively explore the data views, modify analysis parameters to run a new analysis, or add comments and create another automation file for a new round of analysis sharing.

## Discussion

### Reproducible sharing of data analysis

The main purpose of the dChip automation module is to facilitate the sharing of analysis procedures and results when using dChip by reproducing the analysis steps involved. Traditional sharing of results involves transferring data tables and figures as individual files or in an analysis report file. With this automation module, dChip users can easily share data analyses through a data package containing software, raw data, and analysis procedures and parameters that then generates analysis output and figures. This avoids manually preparing multiple data files to be sent to collaborators, who can now use dChip to quickly obtain result files and figures and interactively explore data. New or modified analyses can also be preformed by new users from the shared automation data package. In addition to sharing data and analysis steps in collaborative projects, the dChip user support staff can pinpoint analysis problems or software bugs more efficiently. The learning curve for new dChip users can also be reduced through a demonstrative automation data package.

### Automation data package as supplementary data

Researchers using dChip for microarray analysis can also supplement their publications with a dChip automation data package. Current journal articles often include data files and analysis tables and figures as supplementary material. An interested reader has to follow the methods as described in the paper and locate the software to analyze these data following similar steps. Typically, not every detail of the analysis is provided in the description, leaving a reader unable to reproduce the exact procedures and results. In contrast, a dChip automation data package can allow readers to follow the exact procedures used by the authors and easily modify analysis parameters to further explore a published dataset. This allows datasets and results to be disseminated more rapidly and efficiently. Since the dChip executable file is small, including it in the data package also avoids compatibility issues caused by different versions of dChip and its associated files.

### Relation to reproducible research

Reproducible research has been an active research topic in computer science and statistics [5,7]. It promotes the practice of publishing raw data and analysis programs with an article in a way that the reader can easily reproduce the results presented in the article. A compendium consisting of codes, data, and paper documents has been demonstrated using the R statistical environment [8]. The codes in the compendium can also be modified and run by readers to perform new analyses. Reproducible research is an attractive way to consistently share data and software codes in academic publications and lab documentation. However, its wider adoption requires infrastructure support from multiple computing languages and statistical environments that a research project might use. As discussed above, the dChip automation functions provide a mechanism for sharing reproducible analyses and disseminating supplementary data. We view this new functionality as contributing toward reproducible research. Compared to computer-language-based approaches to reproducible research, the dChip automation module can be more easily used by biologists through menu-driven user interfaces, and it emphasizes reproducible sharing of data and procedures among dChip users rather than creating a readable and executable document embedded with codes. In a compendium using multiple languages and software packages, the dChip software can be called via a shell command to perform a subset of data analyses and visualization according to existing dChip automation step and parameter files.

### Analyzing new or large datasets automatically

Another useful application of the new functionality is to automate dChip to rapidly analyze one or multiple new datasets. Some preprocessing steps are usually similar for different microarray datasets and need minimal user intervention in a preliminary analysis. With the automation module, dChip can be specified to execute the following functions in a sequence: "Open group", "Normalize & Model", "Export expression value", "Filter genes", and "Hierarchical clustering." The required information, such as the directory containing CEL files or the filtering criteria, can be specified through step parameter files. Such an automated analysis would contain functions that a user commonly performs on a dataset and are particularly suitable to run on new datasets generated by a microarray core facility or on multiple datasets of a user. On examining the resulting analysis report file, a user can obtain an initial assessment of data quality and experimental outcomes through array outlier statistics or sample clustering images. With an automation data package, a user can also modify individual steps to perform a refined analysis, such as using a different baseline array for normalization or changing the filtering criteria to obtain more or fewer genes. New users of a microarray core can particularly benefit from such streamlined data analysis services. Other software packages may also export data files and call dChip via shell commands to automatically run certain functions.

## Conclusion

In summary, we have implemented an automation function module in the widely used microarray analysis software dChip. The automation functions reproducibly share data analysis among researchers at the data analysis stage or post-publication via automation data packages containing software, data files, and analysis procedures and parameters. Microarray core facilities may also automate dChip for preliminary analysis and report of new datasets. Similar automation mechanisms can be valuable to the research community when implemented in other genomics and bioinformatics software packages.

## Availability and requirements

Project name: dChip Automation module

Project home page: 

Operating system(s): Windows 2000, XP or above

Programming language: Visual C++ 2005

Source code: Available on request.

License: Software is freely available.

## Authors' contributions

CL designed the software module, implemented and tested the codes, and prepared the manuscript.
